# Study on the Interaction of Polymeric Chemical Additives with Phase Change Materials in Air Lime Renders

**DOI:** 10.3390/polym16081121

**Published:** 2024-04-17

**Authors:** Andrea Rubio-Aguinaga, José María Fernández, Íñigo Navarro-Blasco, José Ignacio Álvarez

**Affiliations:** MATCH Research Group, Department of Chemistry, School of Sciences, University of Navarra, Irunlarrea, 1, 31008 Pamplona, Spain; arubioa@unav.es (A.R.-A.); jmfdez@unav.es (J.M.F.); inavarro@unav.es (Í.N.-B.)

**Keywords:** phase change materials (PCM), lime, render, polymer-based additive, starch, polymeric superplasticizer, adhesion

## Abstract

The interaction of microencapsulated phase change materials (PCMs) with polymeric chemical additives in an air lime binding matrix was studied. These polymer-based additives included an adhesion booster (derived from starch) and a superplasticizer (polycarboxylate ether). Two different PCMs with melting points of 18 °C and 24 °C were assayed. The microcapsules were composed of melamine, with paraffin-based PCM cores. Measurements of zeta potential, particle size distribution, adsorption isotherms, and viscosity analyses were performed to comprehend the behavior of the polymer-based additives within the air lime matrix and their compatibility with PCMs. Zeta potential experiments pointed to the absence of a strong interaction between the lime particles and the microcapsules of PCMs. At the alkaline pH of the lime mortar, the negative charge resulting from the deprotonation of the melamine shell of the microcapsules was shielded by cations, yielding high positive zeta potential values and stable dispersions of lime with PCMs. The polycarboxylate ether demonstrated the ability to counteract the increase in mixing water demand caused by the PCM addition in the lime matrix. The dispersing action of the superplasticizer on the lime particles was seen to exert a collateral dispersion of the PCMs. Conversely, despite the positive values of zeta potential, the addition of the starch-based additive resulted in the formation of large PCM-lime clumps. Air lime renders incorporating 5, 10, and 20% PCMs by weight with various dosages of these chemical additives were experimented with until the optimal formulation for the specific application of the mortars as renderings was achieved. This fine-tuned formulation effectively tackled issues commonly associated with the addition of PCMs to mortars, such as poor adhesion, crack formation, and reduced fluidity.

## 1. Introduction

Presently there is a growing global demand for energy, which poses challenges in the face of escalating climate change and energy supply issues. A key strategy to curb global energy consumption lies in enhancing the energy efficiency of buildings, given that the construction sector accounts for 40% of the final energy requirements in Europe [1,2]. Consequently, recent years have seen revisions in European regulations aimed at reducing the energy demand of new constructions, encouraging the development of highly energy-efficient buildings. However, a significant proportion of existing European buildings, ranging from 35% to 42%, fall into the category of “old” (pre-1960) and exhibit inadequate thermal efficiency, with variations seen across different regions [3]. Within this grouping, historic buildings and monuments that constitute the built heritage hold particular significance, as they typically demonstrate notably low levels of thermal efficiency. This is primarily attributed to the use of inappropriate materials and lenient energy efficiency regulations during their construction [2,3]. Analyses of energy consumption indicate a tendency for consumption to rise with the age of the building. This underscores an opportunity not only to construct new thermally efficient buildings, but also to renovate existing structures in order to enhance their energy efficiency [3].

Numerous methods have been employed to reduce the energy demand of buildings, and one of the most commonly utilized approaches involves modifying the building envelope which is pivotal in determining the overall thermal efficiency of the structure [2,4,5]. However, when it comes to restoring historic buildings, a set of unique challenges arise. This is because extensive modifications must be avoided, and any introduced materials must be highly compatible to preserve the original aesthetic, historic, cultural, spiritual, and social significance [6]. In the last decade, phase change materials (PCMs) have garnered substantial attention in the realm of enhancing the thermal efficiency of buildings, especially for their potential application in latent heat thermal storage (LHTS) systems [7,8,9,10,11,12,13,14]. PCMs are substances capable of storing and releasing latent heat within a specific temperature range through phase transitions, resulting in reduced energy consumption, enhanced thermal comfort by minimizing temperature fluctuations throughout the day, and a reduction and/or shifting of peak loads [15]. The most commonly utilized PCMs are of the solid-liquid type, meaning they can release/store latent heat through processes of solidification and melting [16]. Consequently, during the melting process, the gained heat is retained in the form of latent heat of fusion, and during solidification, this latent heat is released. This leads to a controlled adjustment of the medium’s temperature depending on the ambient conditions [17]. Various methods exist for incorporating PCMs into building materials. These include direct immersion (where the porous building material is immersed in molten PCMs), direct incorporation (where PCMs are mixed directly with the building material), shape stabilization (where PCM is melted and mixed with a polymeric support material), form-stable method (where PCM is trapped in a porous polymeric matrix), macroencapsulation (where panels, spheres, and tubes are filled with substantial amounts of PCMs), and microencapsulation (where PCMs are enclosed in microcapsules) [17]. In this study, the PCMs tested were in the form of microcapsules, which allowed phase changes to occur within the microcapsule without changes in volume or shape. Additionally, this approach facilitated a larger heat transfer area, prevented unwanted interactions and movements of PCMs within the matrix, and effectively prevented leakage [1,16].

Air lime mortar, which is of widespread use and is well known to meet the compatibility requirements for the historic buildings [18], was selected as the matrix material. This matrix is also of interest for modern civil engineering structures [19,20]. The existing literature often concentrates on the incorporation of PCMs, yet tends to overlook the potential alterations in both fresh and hardened properties induced by PCM addition in mortars, including effects such as reduced fluidity, diminished adhesion, crack formation, shrinkage, and a decline in mechanical performance. It is not uncommon to come across references in the literature [21,22,23,24] of increased mixing water to adjust the flow of mortars following the incorporation of PCMs. This leads to, among other things, alterations in material porosity, microstructure, and mechanical characteristics, making it challenging to isolate and understand the unique impact of PCMs addition on mortar properties. This underscores the rationale and novelty of the present work, which is that the use of polymer-based additives can reduce, or even eliminate, the negative effects caused by the addition of phase change materials into an air lime matrix. The use of a plasticizing additive is postulated to improve the workability of the mixes and avoids excess mixing water. Furthermore, an adhesion booster was used as a second additive to improve water retention and adhesion, to avoid cracking and detachment of the lime render once it has been applied on absorbent substrates.

Previous research [25,26,27,28,29] has demonstrated that a polymer derivative of polycarboxylate ether can effectively serve as a superplasticizer, enhancing the workability of fresh mortar augmenting its fluidity. This obviates the necessity for excessive mixing water and allows for fine-tuning the paste’s fluidity to attain a more easily manageable consistency. The molecular structure of these polymeric superplasticizers is characterized by a ‘comb-type’ arrangement, featuring a single primary linear chain with lateral carboxylate and ether groups [30,31,32]. The carboxyl groups, which bear negative charges, generate electrostatic repulsion forces, while the typically hydrophobic and lengthy side chains induce repulsive forces through steric hindrance. The primary mechanism responsible for dispersing particles in these plasticizers is attributed to the latter effect [30].

The use of a polymer starch derivative is simultaneously proposed as an adhesion booster to minimize or prevent cracking and enhance mortar adhesion to various substrates. The mechanism of action for starch polymers lies in their ability to trap water within their structure, leading to a reduction in the amount of unbound water within the mixture and subsequent enhancement of viscosity. Moreover, the lateral chains of the additive can undergo a process of intertwining, further contributing to an increase in viscosity [31].

No effort has been devoted to understanding the action mechanism and the effect of polymeric additives added in air lime matrices containing PCMs microcapsules. Therefore, as a main objective, this study was dedicated to investigating the possible interactions between these two chemical additives and the tested microencapsulated PCMs, as well as their compatibility in an air lime binding matrix. This comprehensive examination provides valuable information, so far not reported in the literature, on the behavior of all components within a lime mortar and on the action mechanism of the polymers. To achieve this objective, zeta potential, particle size distribution, adsorption isotherms, and viscosity studies were carried out.

In a second stage of the current work, microencapsulated PCMs were directly integrated into the air lime matrix at percentages of 5, 10, and 20% by weight of lime. By concurrently introducing the polymers, starch additive, and the polycarboxylate ether derivative, and considering the performance of the additives ascertained during the first stage of the research work, the formulation of PCMs-bearing mortars was fine-tuned. The goal was to not only create materials with enhanced thermal efficiency, but also to produce pastes that are easy to work with, exhibit complete adhesion, and remain free from cracks. To confirm the validity of the proposed formulations and their usefulness in real applications, fresh-state properties of the air lime rendering mortars were studied, including the fluidity, the water retention, and the setting time. A single-layer mortar was applied onto two different substrates (brick and sandstone) visually assessing the adhesion, the formation of cracks, and the shrinkage of the renders. Ultimately, the aim of this second part was to develop a practical building material suitable for real-world applications as a building envelope.

## 2. Materials and Methods

### 2.1. Materials

Hydrated air lime from Calinsa, Pamplona, Navarra, Spain, classified as CL 90-S [33], in powder form, was utilized in the formulation of renders. The average particle size was 10 µm (<10% >50 µm). The lime composition exhibited a CaO content of 96.54%, with major impurities being 1.29% SO_3_, 0.91% MgO, and 0.82% SiO_2_ (measured via X-ray fluorescence, (XRF)). Samples were subjected to XRF analysis utilizing a 4 μm polypropylene filter under a helium atmosphere using the Bruker S2 Puma (Bruker Scientific Instruments, Billerica, MA, USA) apparatus, equipped with an X-ray tube containing a silver anode. Quantitative assessment was conducted by employing the Spectra Results Manager software (Bruker AXS Spectra Elements v2.3). For the aggregate, a very fine limestone with a particle size of 0 to 1 mm, supplied by CTH (Huarte, Navarra, Spain) was used, and its chemical composition measured via XRF was 94.61% CaO and its major impurities were 2.27% SiO_2_, 0.79% Al_2_O_3_, and 0.73% MgO.

Two distinct microencapsulated PCMs in dry powder format supplied by Microtek (Dayton, OH, USA) were utilized. Both microcapsules were composed of melamine, with paraffin-based PCM cores. The difference between the two lay in their respective melting temperatures: one with a melting point of 18 °C (low temperature PCM, referred to as LTPCM) and the other with a melting point of 24 °C (high temperature PCM, referred to as HTPCM). During the mixing process, PCM content by weight of lime (bwol) ranged from 5% to 20% and was directly integrated in bulk into the fresh air lime mortars.

**Figure 1:**
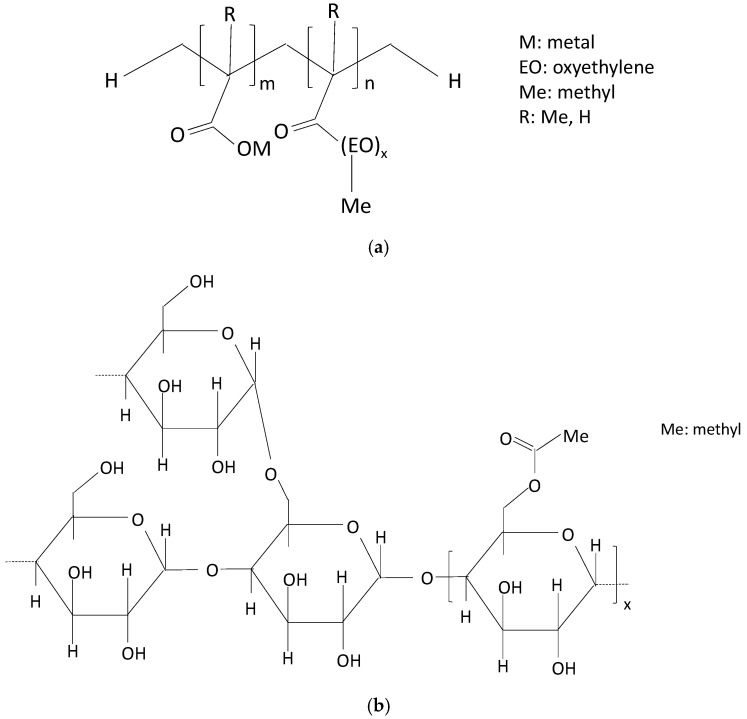
Structures of the chemical additives: (**a**) polycarboxylated-based superplasticizer; (**b**) starch derivative.

Two polymers were used (Figure 1 depicts their chemical structures): a polycarboxylated ether derivative (powder form, MasterCast GT 205 (Master Builders Solutions España S.L.U., Cornellà de Llobregat, Barcelona, Spaim) was launched as a superplasticizer. Its chemical structure consisted of one main linear backbone with side carboxylate and ether groups. According to the supplier, its density at 20 °C ranges from 0.870 to 0.970 g/cm^3^. The average molecular weight, Mw, was 8000 g/mol [30].

The superplasticizer was combined with a starch derivative (powder form, Casaplast KO09 S, Nova Casanova, Canovelles, Barcelona, Spain) to improve adherence. According to the supplier’s data sheet, the adhesion booster is an etherified starch with a high substitution rate that is soluble in cold water with a density of 0.6 g/cm^3^. The additive is categorized as an ultra-high molecular weight polymer of ca. 200 × 10^6^ g/mol. In some previous work, this kind of polymer was fully characterized and the results showed that it was based on amylopectin (ca. 80%) and amylose (ca. 20%) [31].

As a control, a PCM-free mortar (REF-1) was prepared to serve as a basis for comparing PCM performance (the optimization of the formulations of the rendering mortars yielded adjusted mix compositions and are discussed and reported in Section 3.2 below).

### 2.2. Methods

#### 2.2.1. Zeta Potential

Zeta potential measurements were employed to examine the electrostatic forces, whether repulsive or attractive, among particles, providing valuable insights into the stability of various mixtures including PCMs. The measurements were performed by employing a ZetaProbe device from Colloidal Dynamics (Ponte Vedra Beach, FL, USA), utilizing the electro-acoustic technique. Five sets of experiments were conducted:

(a) 5% *wt*/*wt* aqueous lime suspensions with consecutive additions of 1 mL of 5% *wt*/*wt* PCM suspensions in water in order to study the interaction between lime and the PCMs.
(b) Titration with 0.02 M NaOH of 5% *wt*/*wt* PCM suspensions in water with varying amounts of superplasticizer (0%, 0.50%, 0.75%, 1%, and 1.5% by weight of PCM) or starch derivative (0.25%, 0.50%, 0.75%, and 1% by weight of PCM) in order to study the interactions of these chemical additives with the PCMs as a function of pH.
(c) Titration of 5% *wt*/*wt* lime and 5% bwol (by weight of lime) of PCM with 1% *wt*/*wt* aqueous suspensions of superplasticizer or adhesion booster in order to assess the PCM-lime interactions with the chemical additives.
(d) Titration of 5% *wt*/*wt* lime, 5% bwol of PCM and 0.50% bwol of adhesion booster with 1% *wt*/*wt* of superplasticizer (SP).
(e) Titration of 5% *wt*/*wt* lime, 5% bwol of PCM and 0.75% or 1.50% bwol of SP with 1% *wt*/*wt* adhesion booster. These last two sets of experiments were performed for the evaluation of the PCM-lime interactions with the two chemical additives simultaneously.

#### 2.2.2. Particle Size

The particle size distribution was assessed using laser diffraction with a Malvern Mastersizer apparatus (Malvern Panalytical Ltd., Malvern, UK) Three sets of experiments were conducted:

(a) Suspensions containing 5% *wt*/*wt* of PCM in water were prepared, with varying amounts of superplasticizer (0%, 0.50%, 0.75%, 1%, and 1.5% by weight of PCM) or the starch derivative (0%, 0.25%, 0.50%, 0.75%, and 1% by weight of PCM). This aimed to investigate the interaction between the PCMs and these additives.
(b) Another set of experiments involved suspensions comprising 5% *wt*/*wt* of lime, 5% by weight of lime of PCM, and varying amounts of superplasticizer (0%, 0.50%, 0.75%, 1%, and 1.5% bwol) or starch derivative (0%, 0.25%, 0.50%, 0.75%, and 1% bwol). This was performed to examine the interaction between the PCM-lime matrix and the superplasticizer and adhesion booster.
(c) The last set of experiments consisted of aqueous suspensions containing 5% *wt*/*wt* lime, 5% bwol of PCM, 0.50% bwol of adhesion booster and varying amounts of superplasticizer (0%, 0.75%, and 1.50% bwol). This last set was conducted in order to evaluate the simultaneous interaction of the superplasticizer and adhesion booster with the PCM-lime matrix.

#### 2.2.3. Optical Microscopy

The suspensions that were subjected to particle size analysis were also characterized via optical microscopy to visually determine the interaction of the different chemical additives with the PCM-lime system. A Zeiss Axiolab 5 optical microscope (Carl Zeiss Microscopy, Oberkochen, Germany) was used for this purpose.

#### 2.2.4. Viscosity

The impact of the superplasticizer and the starch derivative on the viscosity of PCM-lime pastes was evaluated through viscosity measurements. For the first set of experiments, the pastes were formulated with 50% *wt*/*wt* of lime, 5% of PCM bwol, and varying proportions of superplasticizer (ranging from 0% to 1.5% bwol) or starch (ranging from 0% to 1% by weight of lime). The second set of experiments consisted of pastes with 50% *wt*/*wt* lime, 5% bwol of PCM, 0.50% bwol of starch derivative and varying concentrations of superplasticizer (0%, 0.50%, 0.75%, and 1.50% bwol). Viscosity assessments were conducted using a HAAKE Viscotester 550 (Thermo Fisher Scientific, Waltham, MA, USA), covering a range of 0 to 700 L/s.

#### 2.2.5. Adsorption

Adsorption isotherm curves were generated to assess the affinity of the superplasticizer and the starch for the PCM-lime matrix. Various suspensions were prepared, the first set consisted of 5 g of lime, 0.25 g of PCM, and different quantities of superplasticizer or starch derivative (ranging from 0.0125 g to 0.6000 g). The second set of suspensions contained 5 g of lime, 0.25 g of PCM, 0.025 g adhesion booster and different proportions of superplasticizer (ranging from 0.0125 g to 0.6000 g). In each instance, the suspensions were subjected to magnetic stirring for 30 min and subsequently centrifuged at 8000 rpm for 45 min. The resulting supernatant was filtered using 0.45 µm polytetrafluoroethylene (PTFE) filters, and its absorbance was measured with a Helios Gamma spectrophotometer (Thermo Spectronic, Cambridge, UK). This allowed for the determination of the amount of additive adsorbed onto the binder matrix, calculated as the difference between the initial amount added and the final amount present in the supernatant. The adsorption data was then fitted mathematically to both the Langmuir and Freundlich models for analysis.

#### 2.2.6. Obtaining of the Mortars

**Figure 2:**
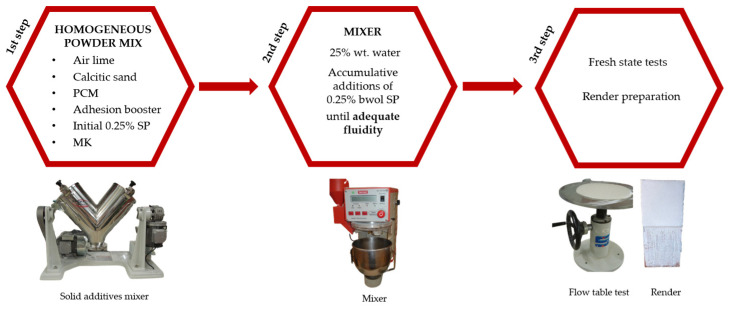
Scheme of the mortar preparation process.

To prepare the fresh mortars, a combination of air lime, sand, starch-based additive, the corresponding PCM, and an initial 0.25% bwol of superplasticizer were mixed using a solid additives mixer BL-8-CA (Lleal, S.A., Granollers, Spain) for 5 min (Figure 2), ensuring a uniform powder blend. In all instances, the weight ratio of binder to aggregate was maintained at 21.7/78.3. Subsequently, 25% *wt*/*wt* of the mixing water was added at low speed over 270 s using a Proeti ETI 26.0072 mixer (Proeti, Madrid, Spain). Gradual increments of 0.25% bwol of superplasticizer were introduced until the desired fluidity (175 ± 20 mm) was achieved and proper adhesion of the monolayer on an absorbent saturated brick surface, based on the judgement of a specialized technician in mortar production and application (Figure 2).

After that, single monolayers of the rendering mortars were applied and studied on two different substrates: brick and sandstone (supplied by DICONA, Pamplona, Navarra, Spain). In both substrates, a single-layer thickness of 0.5 cm was applied and these single-layer renders were kept covered for a period of 15 days to prevent rapid drying of the mortar [34]. Both substrates underwent assessment with respect to their composition, density, and porosity. Concerning composition, determined via XRF (as described before in Section 2.1), the primary constituents for the brick were 47.95% SiO_2_, 26.18% Al_2_O_3_, 9.07% Fe_2_O_3_, and 7.73% CaO, while for the sandstone, they were 80.33% CaO, 8.15% SiO_2_, and 7.44% MgO. Porosity analysis was conducted via mercury intrusion porosimetry (MIP). A Micromeritics AutoPore IV 9500 (Micromeritics Instrument Corporation, Norcross, GA, USA) mercury intrusion porosimeter with a pressure range of 0.0015 to 207 MPa was used, which automatically recorded the pressure, pore diameter, and volume of mercury intrusion, thus determining the pore structure of the material. MIP revealed total porosities of 34.15% for the brick and 20.85% for the sandstone. Open porosity, representing water-accessible porosity and determined using a hydrostatic balance [35], yielded 28.78% for the brick and 15.54% for the sandstone. Lastly, the densities were 1.771 g·cm^−3^ and 2.302 g·cm^−3^ for the brick and sandstone, respectively.

It is important to emphasize that these single coats were prepared specifically for studying the practical application of the mortars as renders, visually observing factors such as adhesion, shrinkage, and crack formation. Thus, a qualitative assessment was conducted to evaluate the level of adhesion and the presence or absence of cracks or fissures. The assessment was based on the following criteria: Degree 3 (complete adhesion to the substrate with no evidence of cracks), Degree 2 (complete adhesion to the substrate with a few and very shallow cracks), Degree 1 (complete adhesion with numerous and shallow cracks), and Degree 0 (poor adhesion with numerous and deep cracks).

#### 2.2.7. Fresh State Tests

Several tests were conducted to characterize the freshly prepared mortars. The flowability of the fresh pastes was assessed through the flow table test, in accordance with the UNE-EN 1015-3 standard [36]. The paste’s density and the proportion of entrapped air were gauged by following the UNE-EN 1015-6 standard [37] and the UNE-EN 1015-7 standard [38], respectively. Water retention capacity was determined by using the UNE-EN 83-816-93 [39]. Lastly, the setting time was evaluated in line with the UNE-EN 1015-9 standard [40].

## 3. Results

### 3.1. Assessment of Compatibility and Interactions of Additives and Lime Particles with PCMs

**Figure 3:**
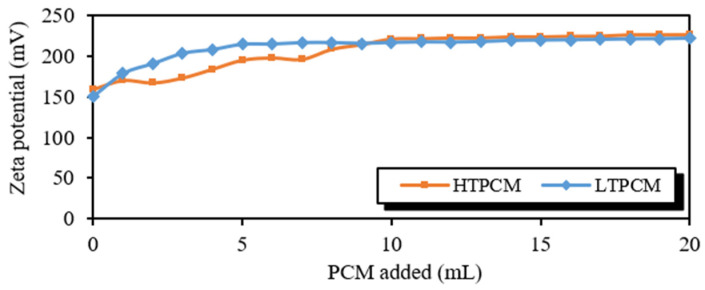
Zeta potential values of PCM titration of lime suspensions.

Firstly, experiments were carried out to evaluate the compatibility of the lime with the different PCMs. Assays conducted on 5% *wt*/*wt* lime aqueous suspensions with regular additions of PCMs (Figure 3) showed asymptotic curves at high and positive zeta potential values. These results suggest the absence of a strong interaction between the lime particles and the microcapsules of PCMs, pointing to a good degree of compatibility between the microcapsules and the lime matrix.

**Figure 4:**
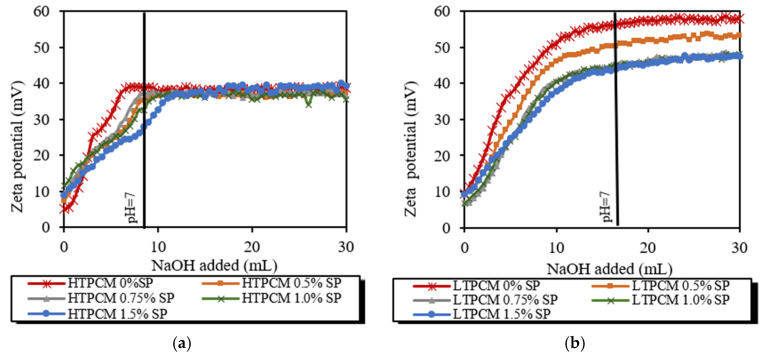

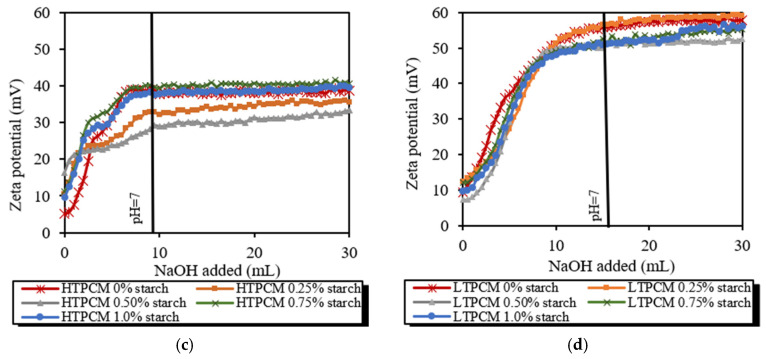
Zeta potential values of 0.02 M NaOH titration of either increasing SP dosages in (**a**) 5% *wt*/*wt* HTPCM, (**b**) 5% *wt*/*wt* LTPCM; or increasing starch dosages in (**c**) 5% *wt*/*wt* HTPCM and (**d**) 5% *wt*/*wt* LTPCM suspensions.

Zeta potential measurements performed on 5% *wt*/*wt* aqueous suspensions of HTPCM or LTPCM with different percentages of superplasticizer are gathered in Figure 4a,b. It was observed how pH clearly affects the charge of the microparticles, probably by deprotonating the melamine. At acidic pH, with little NaOH added, the positively charged protonated melamine is shielded with negative anions responsible for the low zeta potential. As the medium is alkalinized, the negative charge resulting from the deprotonation of the melamine is shielded in the stern layer by cations, responsible for the positive zeta potential, until complete deprotonation, thus reaching an asymptotic curve. Furthermore, the polymeric superplasticizer might employ hydroxyls to deprotonate their carboxyl groups instead of deprotonating the melamine of the microcapsules. This clarified why achieving a high positive zeta potential takes more time, or may not be attained at all, with higher dosages of this chemical additive. In all instances, zeta potential values above 30 mV were obtained at alkaline pH (Figure 4a,b), which indicated a predominance of electrostatic repulsive forces leading to monodispersity with no tendency to agglomerate [41]. Higher zeta potential values can be observed for the LTPCM suspensions indicating higher stability (Figure 4b). The higher zeta potential values for this phase change material can be also related to the higher average diameter of LTPCM (ca. 30 microns) as compared with the HTPCM (ca. 20 microns), which resulted in higher charge surface per microsphere of the PCM. This was confirmed by the requirement of a higher amount of NaOH to fully deprotonate the melamine shield and to reverse the pH from acidic to alkaline values.

The addition of the starch-based additive to any of the 5% *wt*/*wt* water-based PCM suspensions also led to high and positive zeta potential values at alkaline pH levels (Figure 4c,d), particularly for the LTPCM (Figure 4d). The interaction of PCMs with the starch was observed to be more intense. Partial adsorption of the starch on the PCM, especially with HTPCM, could be responsible for these more positive values of the zeta potential at the beginning of the titration. Afterwards, asymptotic, lower values were reached for the intermediate doses, possibly because the interaction or agglomeration between particles did not allow for measurements over the whole surface of the microcapsules. It is noteworthy that the intermediate dose (0.50%) of adhesion booster marked the lowest electrostatic dispersion with the least positive value of the zeta potential (Figure 4c,d).

**Figure 5:**
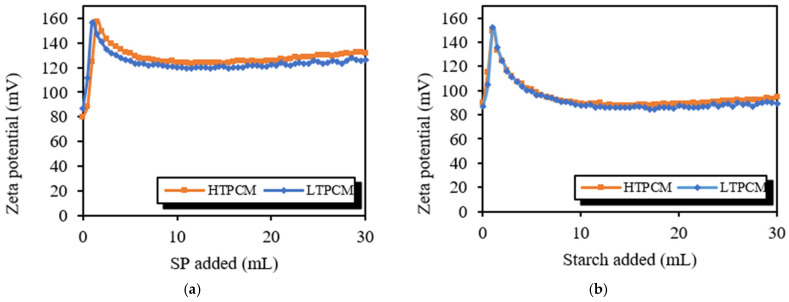
Zeta potential values of 5% *wt*/*wt* lime, 5% bwol PCM: (**a**) titrated with 1% *wt*/*wt* SP and (**b**) titrated with 1% *wt*/*wt* starch.

PCM-lime experiments yielded also high positive zeta potential values (Figure 5). The pastes initially, without any addition of superplasticizer or adhesion booster, showed zeta potential values of 80 to 90 mV due to the positive charge of the portlandite crystals [42]. Upon introducing both polymers, superplasticizer and starch, a substantial rise in zeta potential was detected (up to 150–160 mV), subsequently transitioning to a gradual decline towards lower values. This observation can be attributed to the development of a second adsorption layer, which accounted for the observed trend [42]. In the case of systems containing superplasticizer (Figure 5a), electrosteric dispersion phenomenon accounted for the lesser decrease in zeta potential. Conversely, in starch-containing systems (Figure 5b), an agglomeration phenomenon predominated, resulting in a more significant reduction in zeta potential [31].

**Figure 6:**
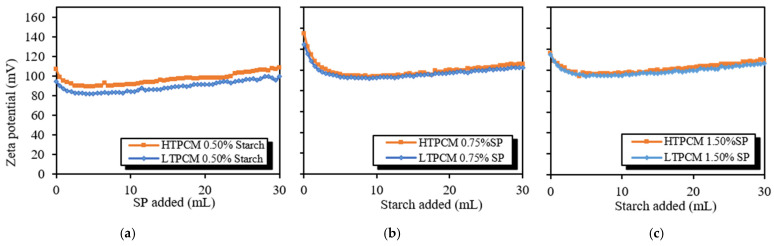
Zeta potential values of 5% *wt*/*wt* lime, 5% bwol PCM: (**a**) initial solution with an additional 0.50% bwol starch titrated with 1% *wt*/*wt* of SP, (**b**) initial solution with an additional 0.75% bwol of SP titrated with 1% *w*/*w* starch, and (**c**) initial solution with an additional 1.50% bwol of SP titrated with 1% *w*/*w* starch.

Zeta potential measurements were carried out on the PCM-lime systems with the simultaneous presence of both the superplasticizer and the starch derivative (Figure 6). First, the PCM-lime system with an initial amount of 0.50% of the starch additive was studied. This system was titrated with superplasticizer to study the interaction of both chemical additives (Figure 6a). The behavior was very similar for the systems with either HTPCM or LTPCM; irrespective of the type of PCM tested, an asymptotic stabilization was observed at high and positive values of zeta potential, indicating the absence of incompatibilities. The lower values of zeta potential observed during the titration with the superplasticizer compared to those reported in Figure 5a could be due to the initial adsorption of starch onto the lime particles, which reduced the number of deprotonated lime particles shielded by calcium ions. Similar results were obtained in experiments conducted in PCM-lime initially including two different percentages of superplasticizer (0.75 and 1.50%) titrated with the starch additive (Figure 6b,c). The disappearance of the maxima observed in Figure 5b was ascribed to the previous adsorption onto lime particles of the superplasticizer, preventing the formation of the second adsorption layer.

**Figure 7:**
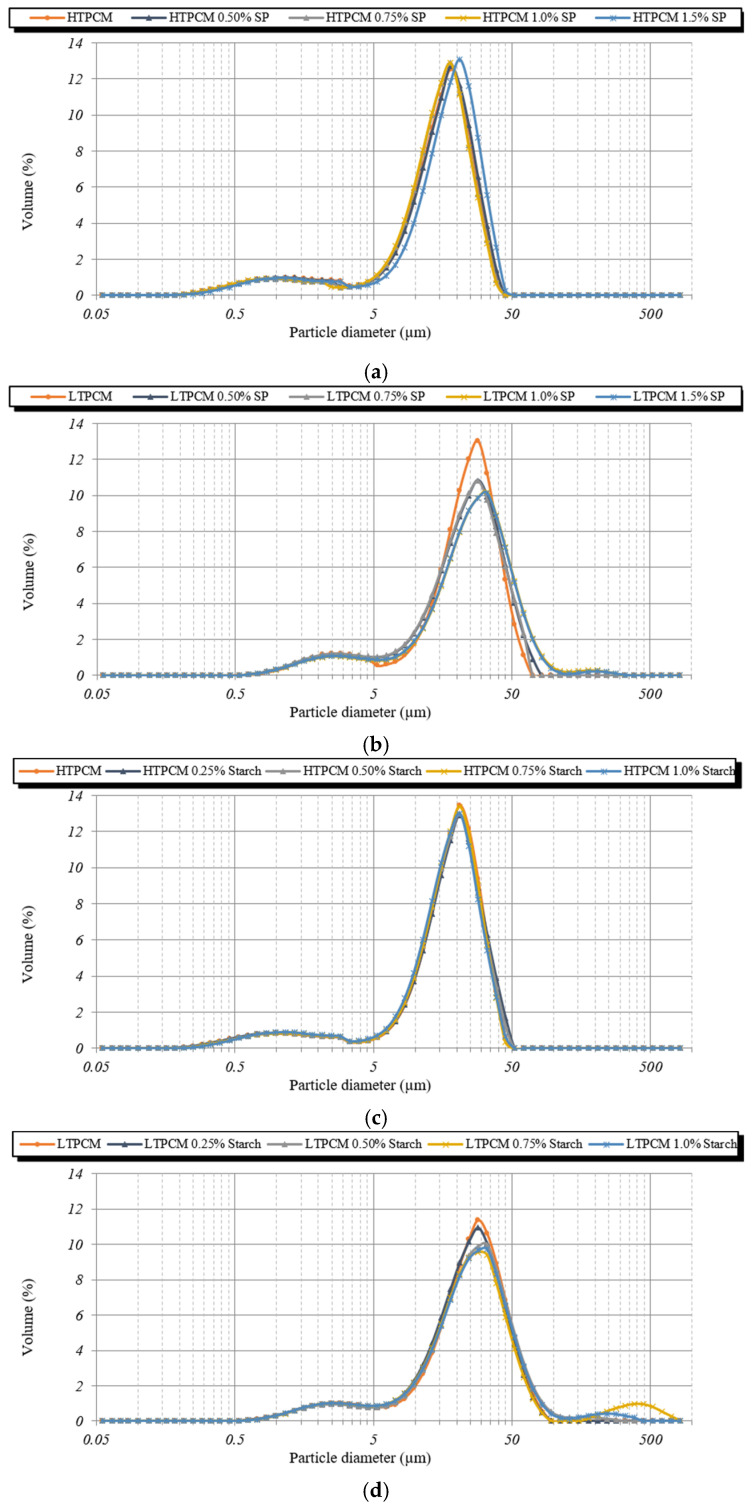
Particle size distribution of 5% *wt*/*wt* lime, 5% bwol of PCM with either increasing superplasticizer dosages in (**a**) 5% bwol HTPCM, (**b**) 5% bwol LTPCM; or increasing starch dosages in (**c**) 5% bwol HTPCM and (**d**) 5% bwol LTPCM suspensions.

Particle size tests were also useful to determine the interactions between the different polymers. Firstly, 5% *wt*/*wt* of PCM suspensions with different dosages of the superplasticizer and the starch additives were analyzed in order to study the interaction between these additives. HTPCM and LTPCM suspensions showed little variation in particle size distribution with increasing superplasticizer percentages (Supplementary Materials, Figure S1a,b). The additions of the starch-based compound also did not result in any changes in the particle size of the aqueous suspensions of both PCMs (Figure S1c,d). These results suggested that the PCMs did not directly interact with either the superplasticizer or the starch derivative. For the PCM-lime suspensions (Figure 7), a more pronounced variation in particle size distribution was detected, indicating interactions between PCM-lime and the additives.

**Figure 8:**
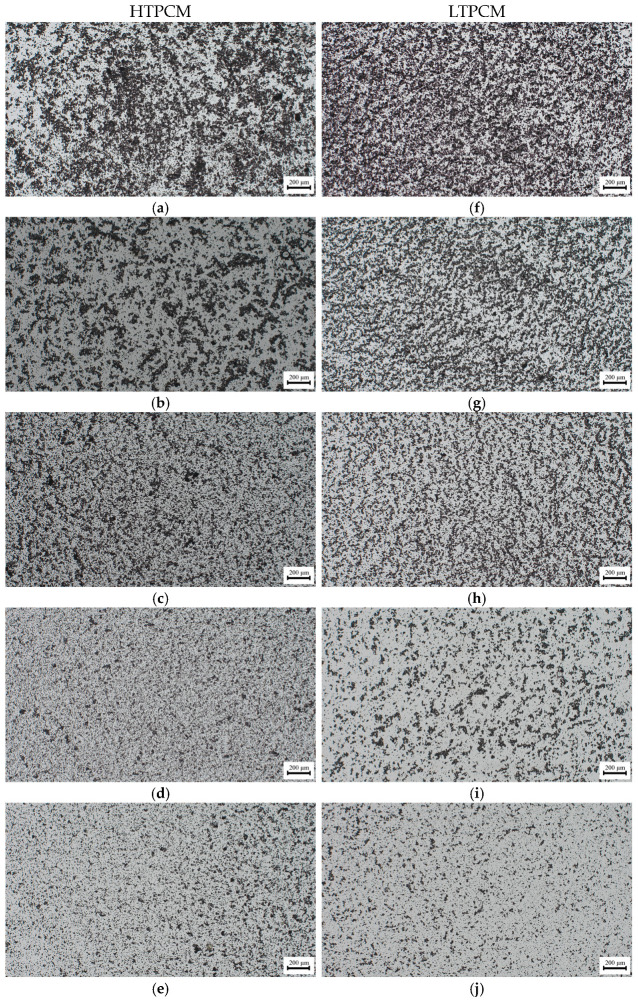
Optical micrographs of PCM-lime suspensions (from top to bottom) with 0, 0.50, 0.75, 1.0, and 1.5% bwol of superplasticizer; (**a**–**e**) corresponds to HTPCM and (**f**–**j**) to LTPCM. All images were captured at 5×.

For the PCM-lime suspensions with superplasticizer dosages (Figure 7a,b), a clear shift to smaller particle sizes was observed, thus exhibiting its dispersing effect [31]. These findings were substantiated through optical microscopy (Figure 8), which demonstrated the deflocculating efficacy of the additive. It prevented the agglomeration of lime particles and induced the dispersion of individual lime particles along with the PCM microcapsules. It was observed how the dispersing action of the superplasticizer on the lime particles influenced in an indirect way the dispersion of the PCMs. The greater the dispersion of the lime particles, the greater the dispersion (absence of agglomerates) of PCMs (see Supplementary Materials, Figure S2), because these PCM microcapsules did not find entanglements of lime particles into which to be retained.

**Figure 9:**
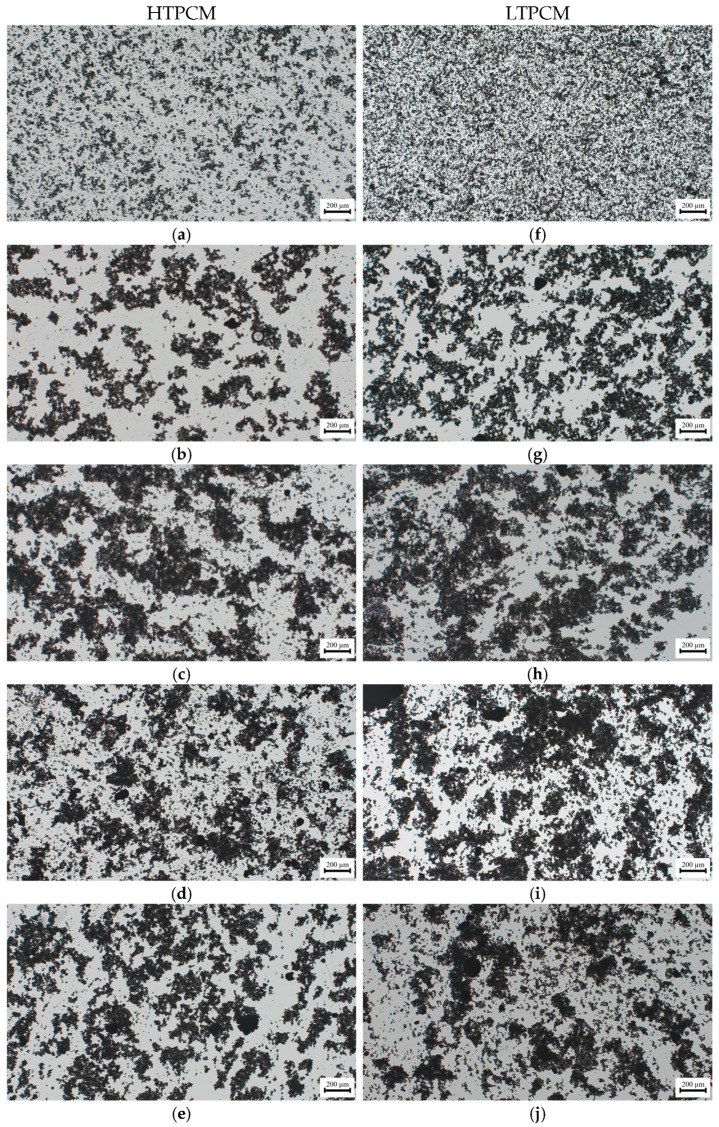
Optical micrographs of PCM-lime suspensions (from top to bottom) with 0, 0.25, 0.50, 0.75, and 1.0% bwol of starch; (**a**–**e**) corresponds to HTPCM and (**f**–**j**) to LTPCM. All images were captured at 5×.

On the other hand, upon addition of the starch-based polymer, a shift towards greater particle sizes was clearly observed (Figure 7c,d), proving the flocculant action of this polymeric additive [31]. This flocculant effect of the adhesion booster was also confirmed via optical microscopy (Figure 9).

**Figure 10:**
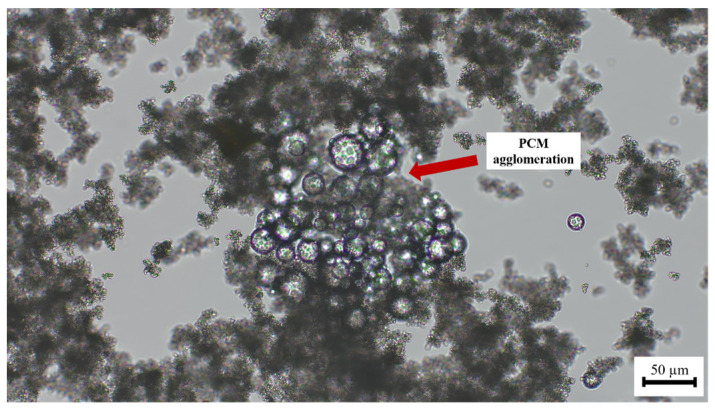
Optical micrograph of LTPCM-lime suspension with 0.50% bwol of starch. Image was captured at 20×.

Large aggregates were observed in the presence of the increasing percentage of the starch derivative (Figure 9). The presence of the chemical additive in the lime matrix was seen to have a strong influence on the distribution of the PCMs. The addition of starch to the PCM-lime system resulted in strong agglomerations of the PCM microcapsules (see Figure 10).

**Figure 11:**
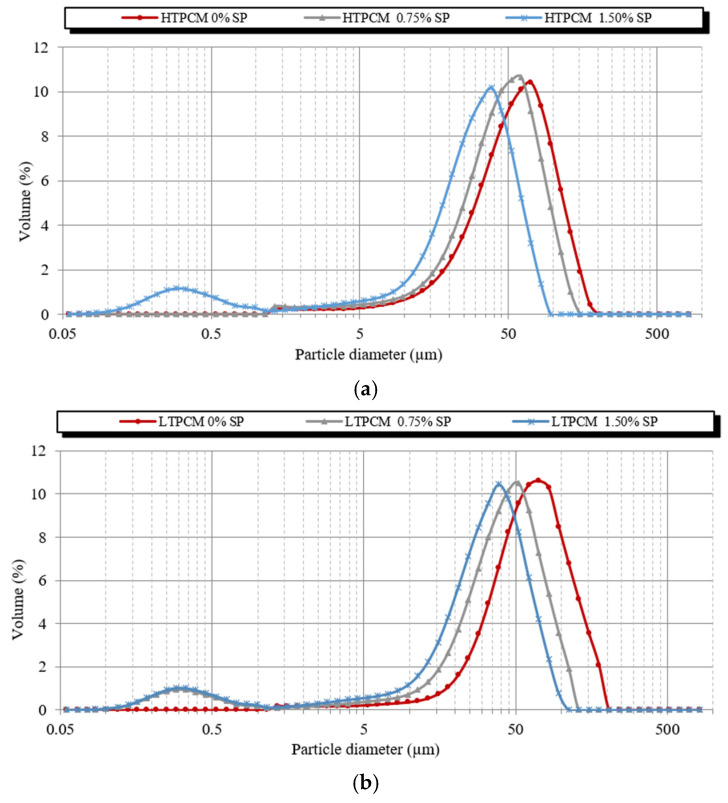
Particle size distribution of 5% *wt*/*wt* lime, 5% bwol of PCM, 0.50% bwol of starch with increasing superplasticizer dosages suspensions: (**a**) HTPCM and (**b**) LTPCM.

After studying the effects of the polymeric additives separately, the particle size distribution of suspensions combining at the same time the PCM-lime matrix with the superplasticizer and the adhesion enhancer (starch derivative) were analyzed. The particle size distribution (Figure 11) shifted towards lower diameter values, thus allowing us to state that the superplasticizing effect of the superplasticizer prevailed over the flocculating action of the starch at the studied dosages. The higher the superplasticizer concentration, the greater the dispersing performance, as confirmed by the smaller particle size.

**Figure 12:**
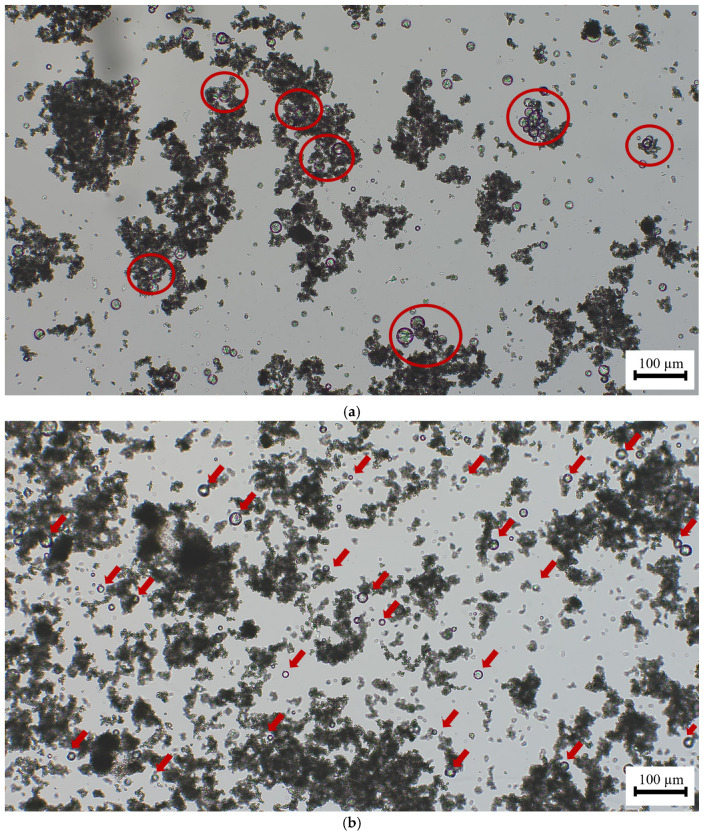
Optical micrographs of suspensions made of 5% *wt*/*wt* lime, 5% bwol of HTPCM, 0.50% bwol of starch with: (**a**) 0% bwol of superplasticizer and (**b**) 0.75% bwol of superplasticizer. Images were captured at 10×. Red circles show agglomerations of PCMs whereas red arrows point to individual isolated PCM microcapsules.

Optical microscopy allowed us to understand the joint performance of the chemical additives. The combination of the polymers and starch with the optimal ratio of the superplasticizer, eliminated the microcapsules’ agglomerations resulting in a homogeneous distribution of the PCMs in the lime matrix (Figure 12a shows the presence of agglomerations of the PCMs while in Figure 12b, by adding the optimal proportion of superplasticizer, these agglomerations disappear, achieving a homogeneous distribution of these microcapsules).

The impact of the polymers on paste viscosity was also evaluated. Viscosity measurements indicated that lime suspensions containing PCM and chemical additives showed a non-Newtonian fluid behavior, specifically depicting shear-thinning characteristics (Supplementary Materials Figure S3 illustrates a viscosity curve exemplifying a reduction in paste viscosity under shear strain). Within this colloidal system, the entanglement of polymeric chemical additives led to relatively high viscosity values when at rest. Nevertheless, when a shear force was applied, polymer chains disentangled, aligning themselves in the direction of the force and causing a decrease in viscosity. Table 1 collects the results for the viscosity of the plain PCM-lime pastes and for the pastes with 0.75% bwol of either SP or starch additive. For pastes with LTPCM, viscosity values were higher than those measured for HTPCM-pastes, in connection with the larger particle diameter of the LTPCM. The reduction in viscosity due to the effective action of the superplasticizer was clearly observed for pastes with the two PCMs. Conversely, the starch addition involved an increase in viscosity, due to the thickening performance of this additive. All these results were in good agreement with the previously reported results on particle size distribution and optical microscopy.

The study of the viscosity values of the PCM-lime pastes with the simultaneous addition of both chemical additives showed that pastes were found to be more viscous with both additives and the presence of superplasticizer increased the fluidity of the pastes (Table 2). This finding could be useful for the practical application of the renders, avoiding the slippage of the mortars.

A comprehensive understanding of the interactions among the additives, demands a clarifying of the adsorption phenomena taking place. To accomplish this, the adsorption onto the PCM-paste of the two additives, namely the superplasticizer and the adhesion enhancer, was examined (main results and isotherm adsorption curves are collected in the Supplementary Materials). In some cases, the obtained results could not be fitted to the Langmuir monolayer model (see Table S1 in the Supplementary Materials). This suggested adsorptions characterized by complex capacities, indicating a multilayer adsorption phenomenon. The adsorption curves for the polycarboxylated ether and the starch-based adhesion booster (depicted in Supplementary Materials, Figure S4) followed the Freundlich mathematical model, as evidenced by the corresponding data (Supplementary Materials, Tables S1 and S2). These findings aligned with the previously discussed zeta potential curves, where the presence of multilayer interactions was established.

**Figure 13:**
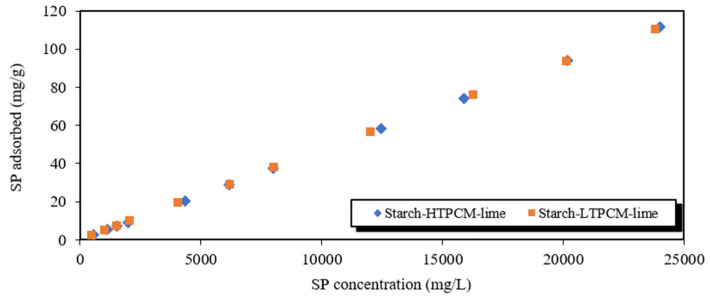
Adsorption isotherm curves of superplasticizer onto starch-PCM-lime.

Adsorption phenomena were also studied in complex systems with the simultaneous presence of both chemical additives (superplasticizer and starch) (Figure 13). 

Table 3 gathers the Langmuir and Freundlich adsorption parameters of the superplasticizer additive onto starch-PCM-lime matrix. It was confirmed that the systems including all the additives, binder particles, and PCMs also followed a multilayer adsorption mechanism. In both cases a strong adsorption of the superplasticizer on the lime based-matrix was observed.

### 3.2. Optimization of the Mix Composition

**Figure 14:**
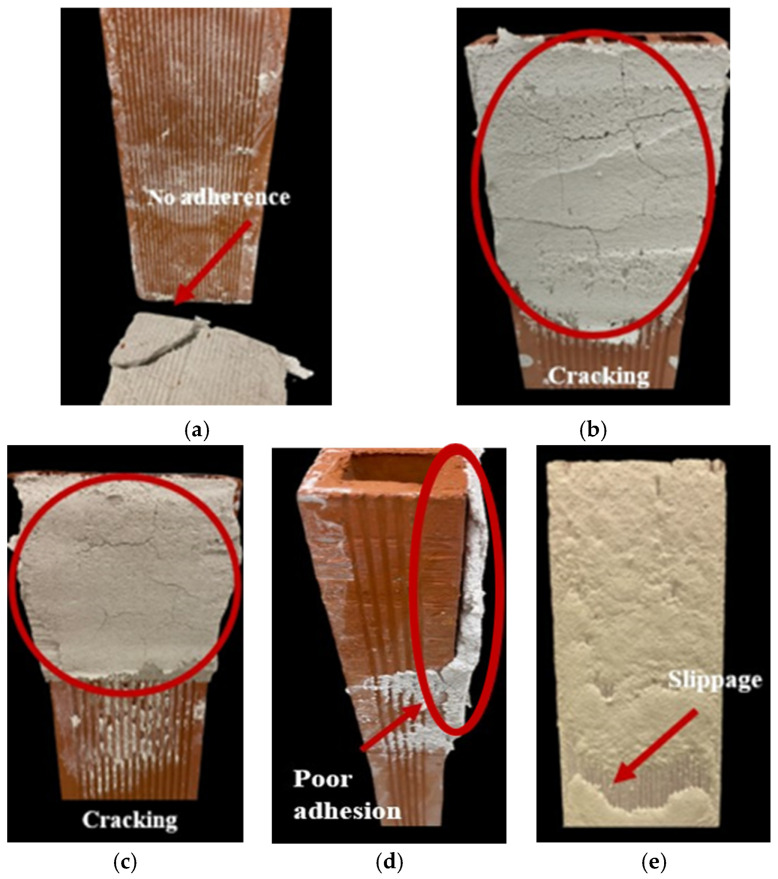
Rendering on saturated brick of mortar: (**a**) containing 10% PCM and 0.45% SP, (**b**) containing 0.35% SP, (**c**) 0.25% starch and 0.40% SP (frontal), (**d**) 0.25% starch and 0.40% SP (side face), and (**e**) 1.25% bwol of SP. All percentages of the additives refer to by weight of lime.

After thorough evaluation of the compatibility and interactions of the polymeric chemical additives and lime particles with the PCMs, the mortar formulation was refined. These preliminary studies on the interactions and stability of systems with the simultaneous presence of the superplasticizer and adhesion enhancer, along with a literature review [27,29,31], allowed for the simplification of the trial-and-error process in establishing the optimal formulation for PCM-bearing mortars intended for application as renders. In this way, the working ranges for each additive were defined, facilitating the initiation of experimentation and expediting the optimization process for the formulation in a more efficient and straightforward manner. As mentioned later in this text, criteria such as adhesion to an absorbent substrate (visually assessed), the spreading capability of the mixture during application, the occurrence of cracks, and the sagging of the monolayer were employed to assess the validity, given their close relevance to the practical application of the material. To assess the efficacy of the starch-based additive, initial render preparations were conducted without the use of an adhesion enhancer, leading to unsatisfactory performance as can be observed in Figure 14a,b, depicting detachment and cracking of the monolayers of the rendering mortars.

**Figure 15:**
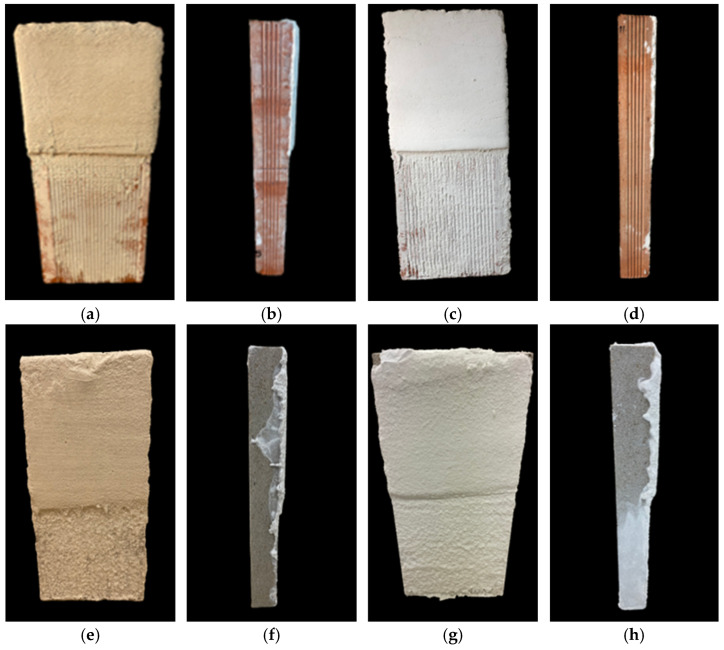
Rendering of mortar on either saturated brick containing: (**a**,**b**) 0.50% starch, 0.75% SP and 5% HTPCM (frontal and side face), (**c**,**d**) 0.50% starch, 0.75% SP and 5% LTPCM (frontal and side face); or saturated sandstone containing (**e**,**f**) 0.50% starch, 0.75% SP and 20% HTPCM (frontal and side face) and (**g**,**h**) 0.50% starch, 0.75% SP and 10% LTPCM (frontal and side face). All percentages of the additives refer to by weight of lime.

As expected, it was evident that the renders required the enhancement of their adhesion. For this reason, the addition of the starch derivative as an adhesion improver in the render formulation was tested. Preliminary compatibility studies (Section 3.1) narrowed down the range of starch ratios to only two percentages, 0.25% (Figure 14c,d) and 0.50% bwol (Figure 15). The lower dosage resulted in a poor adherence and some cracks, as can be seen in Figure 14c,d. However, as pointed out by the zeta potential measurements, the 0.50% ratio was found to be optimal since it successfully prevented crack formation and significantly improved adhesion (Figure 15).

Concurrently, the proportions of the superplasticizer were fine-tuned for each render composition to achieve a manageable texture. Testing a range of superplasticizer percentages from 0.1% to 1.25% bwol revealed that extremes led to either overly fluid and slick renders that slid off the surface (Figure 14e), or excessively dry mixtures that resulted in an unworkable render. As previously documented in the literature [30], this study has reaffirmed the efficacy of polycarboxylate-based superplasticizers for lime mortars. These superplasticizers have demonstrated the ability to counterbalance the impact on the flowability of the fresh mixture caused by the addition of PCMs. Minimal amounts of the tested SP were sufficient to achieve the desired consistency in the renders, without the need for additional mixing water. By regulating fluidity, adhesion, and crack prevention, optimal proportions of adhesion booster and superplasticizer were determined for each render composition containing PCMs. Figure 15 showcases some single-coat mortars with the refined formulations applied onto the brick surface (Figure 15a–d).

Following the attainment of the ideal composition for each render, single-layer mortars were also investigated on a natural substrate, specifically sandstone. This examination resulted in excellent adhesion and the absence of any crack formation, as depicted in Figure 15e–h. Remarkably, optimized renders exhibited exceptional performance despite variances in both the chemical composition and porosity of the substrates employed (Figure 15). The refined formulations have been compiled in Table 4, detailing the proportions of additives utilized for each type, along with the respective percentage of PCM included.

### 3.3. Fresh State Characterization

Tests in the fresh state were conducted on all mortar batches after adjusting the percentages of starch and superplasticizer for each render. Table 5 includes values for slump, setting time, paste density, entrained air, and water retentivity. A qualitative evaluation of the rendering comprising four degrees was also included: Degree 3 represented complete adhesion without cracks, Degree 2 indicated complete adhesion with a few shallow cracks, Degree 1 denoted complete adhesion with numerous shallow cracks, and Degree 0 signified poor adhesion with numerous deep cracks.

In broad terms, as outlined in Table 5, the inclusion of any PCM at varying percentages does not lead to a significant modification in stiffening time, entrained air, paste density, or water retentivity. This was attributed to the concurrent use of diverse polymeric additives. All mortars demonstrated complete adhesion to the substrate, with minimal to no occurrence of cracking. Furthermore, all consistencies were suitable for their intended application as a render. The introduction of microencapsulated PCMs did not have a severe impact on the paste’s consistency, thanks to the simultaneous adjustment of the polymers, adhesion booster, and superplasticizer. In every instance, the paste’s fluidity (ranging from 161 mm to 189 mm) allowed for easy workability and spreadability. These findings support the compatibility and stability of the chemical additives within the PCM-lime system, as demonstrated earlier (refer to Section 3.1). Thus, it underscores the significance of thorough formulation optimization to design mortars tailored to their intended end-use.

## 4. Conclusions

This research conducted a comprehensive investigation into the interactions between two polymeric chemical additives (polycarboxylate ether and starch) and microencapsulated PCMs (differing primarily in their melting temperatures, with one at 18 °C and the other at 24 °C) within the air lime matrix. The compatibility of the PCMs with the lime matrix was studied. Results from zeta potential experiments on lime-PCM indicated a lack of strong interaction between lime particles and PCM microcapsules, suggesting a high level of compatibility between the microcapsules and the lime matrix. Interaction experiments between PCM and superplasticizer resulted, at alkaline pH, in zeta potential values above 30 mV indicating a predominance of repulsive electrostatic forces leading to monodispersity with no tendency to agglomeration. Higher zeta potential values were obtained from experiments studying the behavior of suspensions with PCM and starch, elucidating a more intense interaction. In addition, the experiments with PCM-lime also yielded high positive zeta potential values (80–150 mV). In the case of the superplasticizer-containing systems, the phenomenon of electrosteric dispersion was ascertained. In contrast, the starch-bearing systems were dominated by an agglomeration phenomenon. Experiments of the PCM-lime systems with the simultaneous presence of the superplasticizer and of the starch also showed high zeta potential values indicating the absence of incompatibilities.

By means of the particle size tests, together with optical microscopy, an indirect interaction between the polymer-based additives and the PCMs was elucidated. The action mechanism of the additives took place through their interaction with lime particles. Specifically, the distribution and dispersion of the microcapsules were found to be dependent on the distribution and dispersion of lime particles, which, in turn, were influenced by the accurate dosing of the chemical additives. For lime-PCM systems (LTPCM and HTPCM), an increase in particle size was generally observed with higher starch dosage, as starch tends to agglomerate. Conversely, the superplasticizer had the opposite effect, reducing particle size due to its dispersing action. The adsorption phenomena of the polymeric additives on PCM-lime conformed to the Freundlich-type multilayer model and remained consistent irrespective of the PCM’s melting temperature. Viscosity measurements were primarily affected by the PCM in the paste, a parameter dominated by differences in average diameter (approximately 30 μm for LTPCM and 20 μm for HTPCM). Nevertheless, the impact of the chemical additives followed the expected pattern, with the starch derivative increasing paste viscosity and the superplasticizer enhancing fluidity.

These findings highlight the importance of studying diverse interactions among different polymer-based additives in mortar to design an optimal material for its intended application. Preliminary investigations into compatibility and stability guided the ratios for each additive, and through performance assessments (crack formation and adhesion) on absorbent substrates, the optimal formulation was achieved. This enabled the production of renders containing 5%, 10%, or 20% by weight of PCM with full adhesion that were devoid of cracks, easy to work with, and spread, and that alleviated common issues associated with PCM addition. Fresh state tests indicated that, post-formulation optimization, mortars containing any type or proportion of PCM exhibited very similar properties to those without this additive, rendering them suitable for real application in buildings. Subsequent studies will focus on examining the thermal performance of these optimized renders.

## Figures and Tables

**Table 1:** Viscosity coefficients at a shear rate of 2.7 s^−1^ for plain lime pastes and lime pastes with just one additive.

	SP (% bwol)	Apparent Viscosity (mPa·s)	Starch (% bwol)	Apparent Viscosity (mPa·s)
HTPCM	0.00	422	0.00	422
	0.75	198	0.75	10,720
LTPCM	0.00	2690	0.00	2690
	0.75	1644	0.75	11,850

**Table 2:** Viscosity coefficients at a shear rate of 2.7 s^−1^ for lime pastes with 0.5% of starch and SP (0 or 0.5%).

	Starch (% bwol)	SP (% bwol)	Apparent Viscosity (mPa·s)
HTPCM	0.50	0.00	11,660
	0.50	0.50	4763
LTPCM	0.50	0.00	3297
	0.50	0.50	2275

**Table 3:** Results of adsorption isotherms onto starch-PCM-lime suspensions of the superplasticizer: Langmuir and Freundlich adsorption parameters.

	Langmuir			Freundlich		
	qm (mg/g)	b (L/mg)	R^2^	K	1/n	R^2^
HTPCM	6591.6	7.16 × 10^−7^	0.8133	0.00486	0.9958	0.9999
LTPCM	4578.5	1.03 × 10^−6^	0.9673	0.00494	0.9941	0.9999

Notes: qm: maximum sorption capacity. b: The Langmuir constant. K, 1/n: the Freundlich constants. R^2^: correlation coefficient of the linear regression.

**Table 4:** Percentages of PCM, SP and starch derivative of the renders.

Render	LTPCM (% bwol)	HTPCM (% bwol)	SP (% bwol)	Starch (% bwol)
REF-1	-	-	0.60	0.50
HTPCM-1	-	5	0.75	0.50
HTPCM-2	-	10	0.75	0.50
HTPCM-3	-	20	0.75	0.50
LTPCM-1	5	-	0.75	0.50
LTPCM-2	10	-	0.75	0.50
LTPCM-3	20	-	0.75	0.50

**Table 5:** Results of the fresh state tests of PCM-free and PCM-bearing renders.

Render	Slump (mm)	Stiffening Time (min)	Paste Density (kg/L)	Entrained Air (%)	Water Retentivity (%)	Qualitative Evaluation of Rendering (0–3)
REF-1	182	1157	1.94	4.3	95.9	3
HTPCM-1	177	1211	1.83	6.3	95.2	3
HTPCM-2	175	1392	1.85	7.0	93.1	3
HTPCM-3	161	1432	1.79	7.0	92.2	3
LTPCM-1	178	1119	1.95	2.2	93.5	3
LTPCM-2	189	1580	1.93	1.7	92.3	3
LTPCM-3	183	1769	1.89	1.1	93.1	2

## Data Availability

The datasets presented in this article are not readily available because the data are part of an ongoing study.

## Supplementary Materials

The following supporting information can be downloaded at: https://www.mdpi.com/article/10.3390/polym16081121/s1, Figure S1: Particle size distribution of 5% *wt*/*wt* PCM with either increasing superplasticizer dosages in: (a) 5% *wt*/*wt* HTPCM, (b) 5% *wt*/*wt* LTPCM; or increasing starch dosages in (c) 5% *wt*/*wt* HTPCM and (d) 5% *wt*/*wt* LTPCM suspensions; Figure S2: Optical micrograph of HTPCM-lime suspension with 1% bwol of superplasticizer. Most of the microcapsules of PCMs are denoted with red arrows to identify their dispersion. Image captured at 10×; Figure S3: Viscosity measurements of 50% *wt*/*wt* lime, 5% bwol of HTPCM and 0.75% bwol of SP; Figure S4: Adsorption isotherm curves onto PCM-lime of: (a) superplasticizer and (b) starch; Table S1: Results of adsorption isotherms onto PCM-lime suspensions of superplasticizer: Langmuir and Freundlich adsorption parameters; Table S2: Results of adsorption isotherms onto PCM-lime suspensions of adhesion booster: Langmuir and Freundlich adsorption parameters.
