# Supplementary material for: Study on the Interaction of Polymeric Chemical Additives with Phase Change Materials in Air Lime Renders
Source: Polymers (Basel). 2024 Apr 17;16(8):1121. doi: 10.3390/polym16081121 (PMC11053762; doi:10.3390/polym16081121)
Supplement: Supplementary file 1 [file polymers-16-01121-s001.zip › polymers-2958842-supplementary.pdf]

SUPPLEMENTARY MATERIAL

Study on the interaction of polymeric chemical additives with phase change materials in air lime renders

A. Rubio-Aguinaga[a], J.M. Fernández[a], I. Navarro-Blasco[a], J.I. Álvarez[a*]

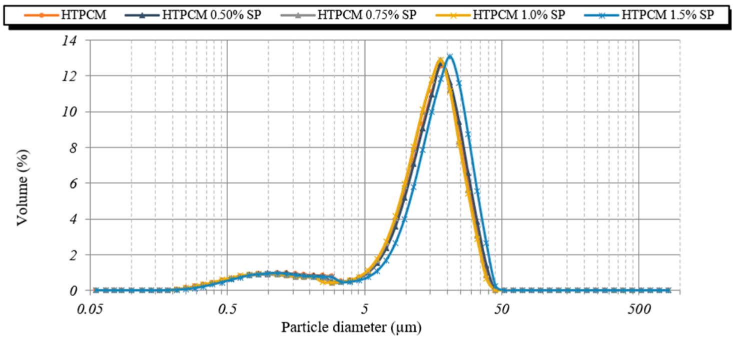

(a)

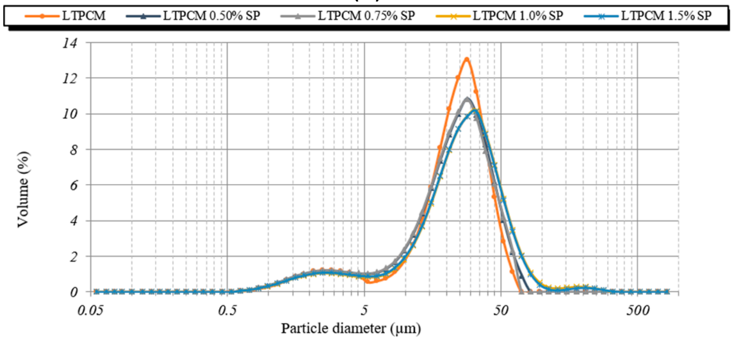

(b)

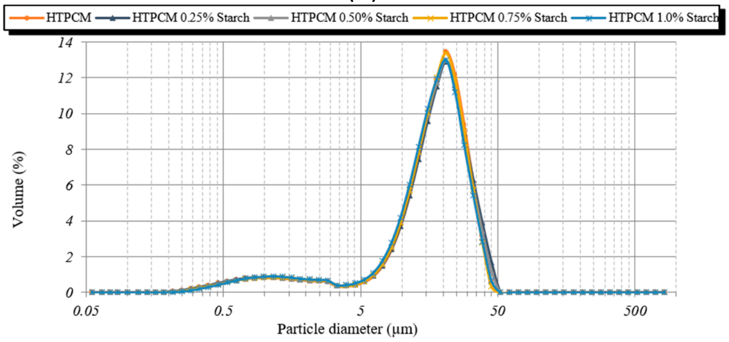

(c)

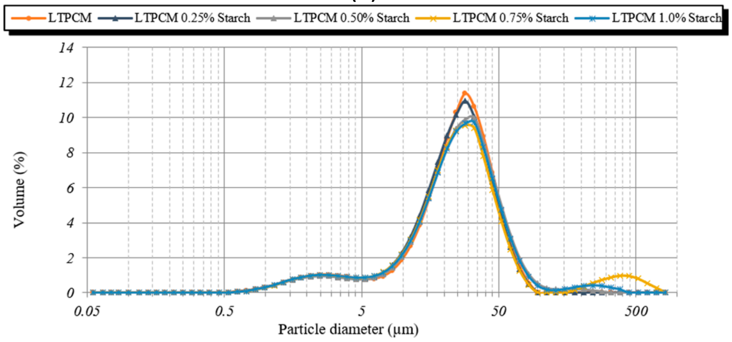

(d)

Figure S1: Particle size distribution of 5% wt/wt PCM with either increasing superplasticizer dosages in: (a) 5% wt/wt HTPCM, (b) 5% wt/wt LTPCM; or increasing starch dosages in (c) 5% wt/wt HTPCM and (d) 5% wt/wt LTPCM suspensions.

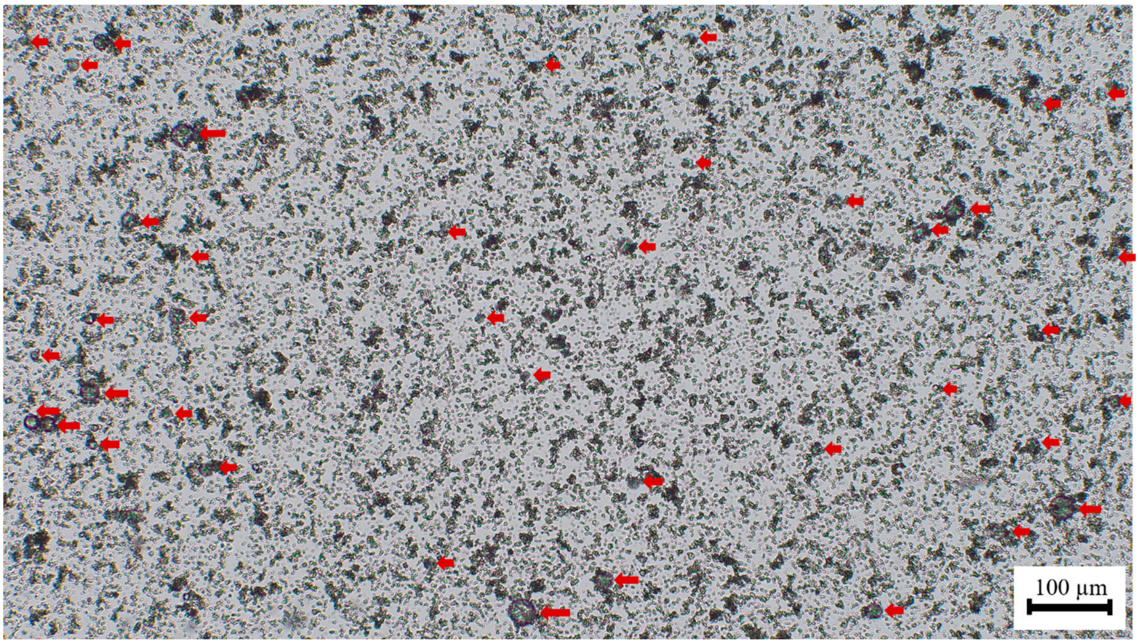

Figure S2: Optical micrograph of HTPCM-lime suspension with 1% bwol of superplasticizer. Most of the microcapsules of PCMs are denoted with red arrows to identify their dispersion. Image captured at 10x.

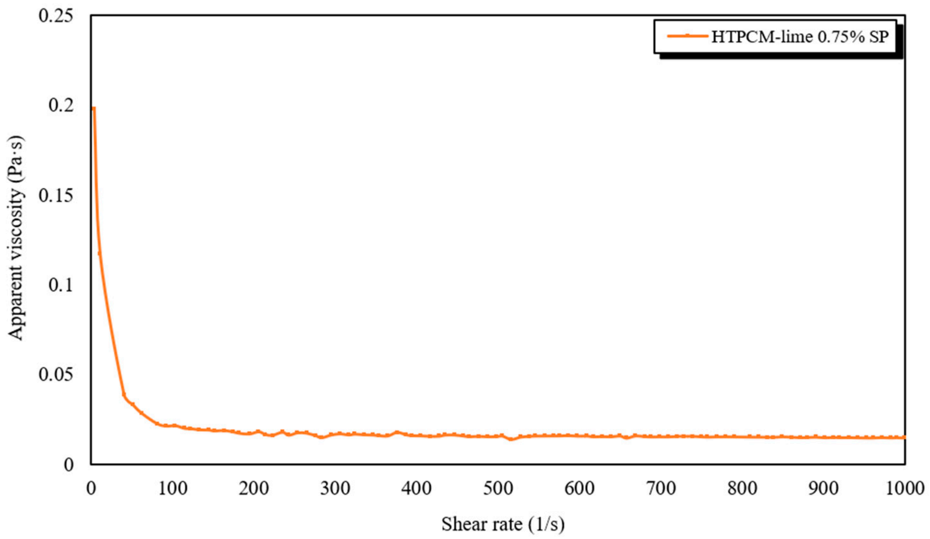

Figure S3: Viscosity measurements of 50% wt/wt lime, 5% bwol of HTPCM and 0.75% bwol of SP.

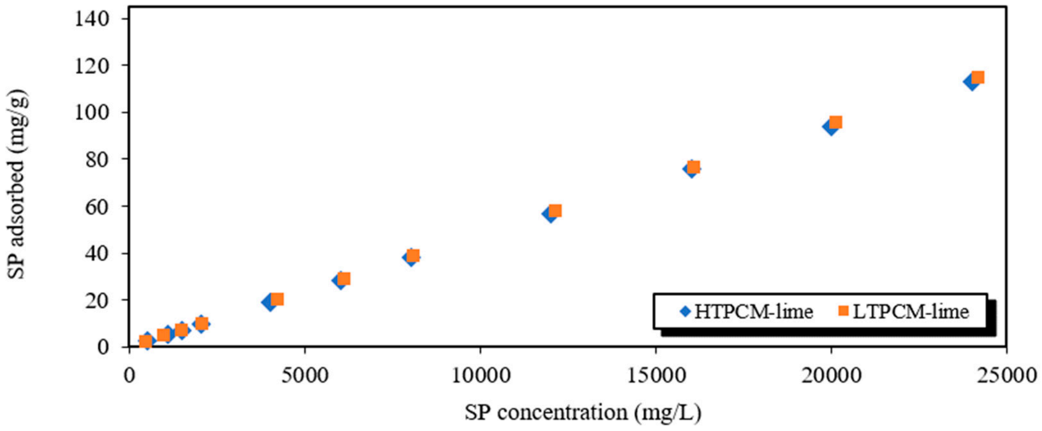

(a)

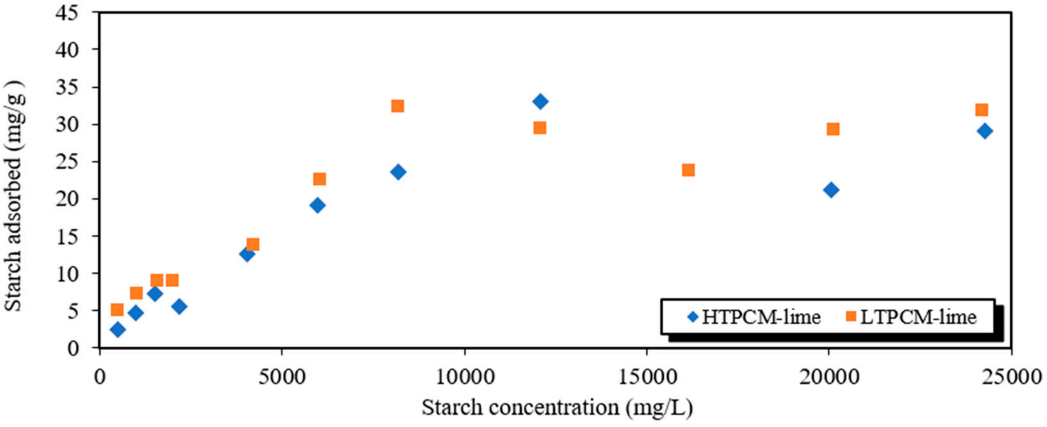

(b)

Figure S4: Adsorption isotherm curves onto PCM-lime of: (a) superplasticizer and (b) starch.

Table S1: Results of adsorption isotherms onto PCM-lime suspensions of superplasticizer: Langmuir and Freundlich adsorption parameters.

	Superplasticizer					
	Langmuir			Freundlich		
	qm (mg/g)	b (L/mg)	R^2	K	1/n	R^2
HTPCM	10762.5	$4.41 \cdot 10^{-7}$	0.7024	0.00485	0.9970	0.9999
LTPCM	-	-	-	0.00464	1.0021	0.9999

Notes: qm: maximum sorption capacity. b: the Langmuir constant. K, 1/n: the Freundlich constants. R^2*: correlation coefficient of the linear regression.*

Table S2: Results of adsorption isotherms onto PCM-lime suspensions of adhesion booster: Langmuir and Freundlich adsorption parameters.

	Adhesion booster					
	Langmuir			Freundlich		
	qm (mg/g)	b (L/mg)	R^2	K	1/n	R^2
HTPCM	40.2	$1.33 \cdot 10^{-4}$	0.6727	0.03598	0.7003	0.8811
LTPCM	36.1	$2.27 \cdot 10^{-4}$	0.8734	0.22099	0.5058	0.9213

Notes: qm: maximum sorption capacity. b: the Langmuir constant. K, 1/n: the Freundlich constants. R^2*: correlation coefficient of the linear regression.*
